# Classical phenylketonuria presenting as maternal PKU syndrome in the offspring of an intellectually normal woman

**DOI:** 10.1002/jmd2.12384

**Published:** 2023-07-25

**Authors:** Malak Ali Alghamdi, Anne O'Donnell‐Luria, Naif A. Almontashiri, Wajeih Y. AlAali, Hebatallah H. Ali, Harvey L. Levy

**Affiliations:** ^1^ Harvard Medical School Boston Massachusetts USA; ^2^ Medical Genetic Division, Pediatric Department College of Medicine, King Saud University Riyadh Saudi Arabia; ^3^ Program in Medical and Population Genetics Broad Institute of MIT and Harvard Cambridge Massachusetts USA; ^4^ Division of Genetics and Genomics Boston Children's Hospital Boston Massachusetts USA; ^5^ Center for Genetics and Inherited Diseases (CGID) Taibah University Madinah Saudi Arabia; ^6^ Dr. Sulaiman Al Habib Medical Group Arryan Hospital Riyadh Saudi Arabia; ^7^ Research Center, College of Medicine King Saud University Riyadh Saudi Arabia

**Keywords:** maternal PKU syndrome, neonatal deaths, phenylalanine hydroxylase, recurrent pregnancy loss, variable expressivity

## Abstract

Phenylketonuria (PKU) is an autosomal recessive inborn error of metabolism resulting from a deficiency of phenylalanine hydroxylase (PAH). If untreated by dietary restriction of phenylalanine intake, impaired postnatal cognitive development results from the neurotoxic effects of excessive phenylalanine (Phe). Signs and symptoms include severe intellectual disability and behavior problems with a high frequency of seizures and variable microcephaly. Maternal PKU syndrome refers to fetal damage resulting in congenital abnormalities when the mother has untreated PKU during pregnancy. Here, we report an intellectually normal 32‐year‐old female who presented with recurrent pregnancy loss and two neonatal deaths with congenital heart disease, microcephaly, intrauterine growth restriction, and respiratory distress. She was diagnosed with PKU through exome sequencing performed for carrier testing with a homozygous pathogenic variant in the PAH gene, c.169_171del, p.(Glu57del) that is associated with classical PKU. Consistent with the genetic finding, she had a markedly increased plasma phenylalanine concentration of 1642 μmol/L (normal <100). This case demonstrates that recurrent pregnancy loss due to untreated maternal PKU may present as an initial finding in otherwise unsuspected classical PKU and illustrates that extreme degrees of variable expressivity may occur in classical PKU. Moreover, this case illustrates the value of genomic sequencing of women who experience recurrent pregnancy loss or neonatal anomalies.

**Synopsis**

Recurrent pregnancy loss and neonatal deaths due to untreated maternal PKU syndrome may present as an initial finding in intellectually normal women diagnosed with classical PKU, indicating that variable expressivity may occur in classical PKU.

## 1 INTRODUCTION

Phenylketonuria (PKU) is an autosomal recessive inborn error of metabolism resulting from a deficiency of phenylalanine hydroxylase (PAH; 612349). PAH deficiency presents as a phenotypic spectrum of severity; most severely affected are individuals with complete enzyme deficiency whose untreated blood phenylalanine (Phe) levels are typically >1200 μmol/L; this phenotype is consistently termed “classical PKU.”^1^

Since the initiation of NBS in many countries, almost all cases of PAH deficiency are diagnosed following a positive newborn screening test for elevated blood spot Phe.^2^ This results in significant benefits for affected individuals through the ability to offer early therapy.^3^ Dietary therapy involving Phe restriction with severe protein restriction and supplementation with reduced or Phe‐free amino acid mixtures (medical foods, “formulas”) is effective in preventing severe cognitive impairment associated with untreated classical PAH deficiency.^4^, ^5^, ^6^ Over time, subtle intellectual and neuropsychiatric issues may manifest even with treatment,^7^, ^8^, ^9^, ^10^ especially if strict metabolic control is not maintained. These features may be reversible in the early stages of loss of control but if poor metabolic control persists, they become much more difficult to reverse.^11^, ^12^ Nevertheless, even severely intellectually disabled adults with late‐diagnosed PAH deficiency may show improvements in challenging behavior with lowering of blood Phe levels.^13^

Pregnancy presents a particular problem in women with PKU. High levels of Phe are toxic to the brain as well as the heart in the developing fetus resulting in the maternal PKU (MPKU) syndrome.^14^, ^15^, ^16^ Here, we present a case of a woman with a classical PKU genotype and biochemical profile in whom the presenting features were recurrent pregnancy losses and two neonatal deaths.

## 2 CARE REPORT

Mr. AZ and Mrs. RZ (II‐1, II‐2) are a healthy Palestinian couple married for the last 10 years. RZ is 33 years old healthy female (II‐2) who competed her bachelor's degree in education and AZ is 35‐year‐old male (II‐1) who completed his bachelor in science. The couple presented to genetic clinic with a history of recurrent pregnancy losses; two previous pregnancies resulted in early miscarriages and two pregnancies resulted in neonatal deaths with congenital heart disease due to the hypoplastic left heart syndrome, microcephaly, and respiratory distress. They have one living offspring, a 6‐year‐old son, SZ, (III‐2) with mild intellectual impairment, behavioral issues, and autism spectrum disorder. He was evaluated by the neurology and genetic services. Metabolic and genetic testing results were unremarkable. His brain Magnetic Resonance Imaging (MRI) and computed tomography (CT) scans were normal.

**FIGURE 1:**
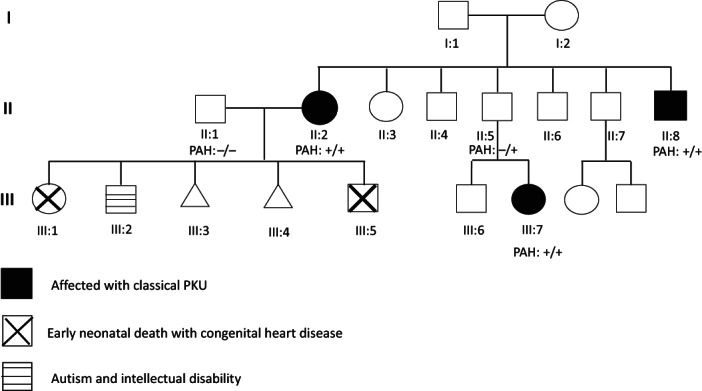
Family pedigree.

Family history revealed that RZ has one brother, FZ, with classical PKU (II‐8). He was diagnosed early and well controlled on medical formula and Kuvan. Also, her niece, WZ, is 2 years old (III‐7) diagnosed with PKU through NBS and is doing well on treatment (Figure 1).

Physical examination of RZ was unremarkable; in particular, there were no dysmorphic features, no typical physical characteristics of PKU for example, fair skin, blond hair, blue eyes, and eczema. Her head circumference was 54.5 cm (57th centile). She was entirely normal on full neurological and psychological assessments with no abnormalities in her intellectual and neuropsychological function. She had no history of learning difficulty or behavioral problems at school.

Following initial assessment including parental chromosomal analysis and maternal hematological and autoimmune work up, they were referred to a Medical Genetic clinic. Due to the recurrence of a similar phenotype in two neonates, exome sequencing was performed on both parents. After excluding all other causes of recurrent pregnancy loss, we proceeded to duo parental exome sequencing of AZ and RZ to exclude their carrier status of pathogenic or likely pathogenic variants that were shared between these parents that could result in an affected offspring with a cardiac phenotype.

Exome sequencing revealed that the mother, RZ, is homozygous for a three‐basepair in‐frame deletion variant c.169_171del, p.Glu57del in the *PAH* gene, consistent with the genetic diagnosis of autosomal recessive PKU. Plasma amino acid testing showed a Phe level in the classic range at 1642 μmol/L and a second level of 1492 μmol/L obtained a few days later (Table 1).

**TABLE 1:** Biochemical results for RZ showing elevated phenylalanine.

Test	Result	Unit	Reference range
Phenylalanine	1642	μmol/L	40–74
Phenylalanine (the repeat)	1492	μmol/L	40–74
Tyrosine	42	μmol/L	38–96

No other clinically relevant sequencing variants or copy number variations related to the described phenotype were identified. Chromosomal analyses had been performed on the parents and the neonate from the second affected pregnancy and they were unremarkable. The brother and niece of RZ each are homozygous for the same pathogenic variant and affected with PKU. Exome sequencing in their son, RZ, disclosed no clinically relevant variant so he is not homozygous for this variant but his carrier status was not reported.

Directly after the establishment of the diagnosis, the patient was treated with a Phe‐restricted diet. The addition of sapropterin was requested. Phe concentrations on treatment measured every 2 weeks ranged 300–500 μmol/L. Three months after initiation of the diet, she became pregnant. During pregnancy her Phe levels were monitored weekly and the Phe level was maintained at 120–360 μmol/L. Ultrasonographic examinations during the first trimester showed no anatomical anomalies.

## 3 DISCUSSION

PAH deficiency shows a broad phenotype spectrum ranging from classic PKU to mild hyperphenylalaninemia (HPA), depending on the residual enzymatic activity.^17^ The c.169_171del in‐frame deletion variant that was identified in our patient has been identified in at least three individuals with phenotypes of mild to classic PKU.^18^, ^19^ It has been detected in trans with pathogenic variants c.1208C>T in a proband with mild PKU and with c.1066‐11G>A in a proband with BH4‐responsive, mild PKU.^18^, ^19^, ^20^ Additionally, similar to this report, the c.169_171del homozygous *PAH* variant was reported in a 20‐year‐old female from a consanguineous family who was diagnosed at 18‐months of age with classical PKU (Phe level was 1152 μmol/L).^21^ In contrast to the clinically normal mother in our report, this woman had hypotonia, developmental delay, microcephaly, epilepsy, and MRI abnormalities consistent with the untreated classic PKU phenotype.

Approximately in 80%–90% of PAH deficient patients, there is a strong correlation between the genotype and biochemical phenotype, also it must be stated that the clinical phenotype in the untreated state follows with intellectual ability ranging from profound intellectual disability in PKU to normality in mild hyperphenylalaninemia (MHP).^22^ Nevertheless, there have been reports of rare individuals with untreated PKU who are intellectually normal or near normal.^23^ How these individuals have escaped the deleterious brain effect of elevated Phe concentrations is unknown despite attempts to identify modifier genes. One study has hypothesized that a variant in the *SHANK* gene could be protective and modify the effect on cognitive development in PKU but this has not been confirmed.^24^

Although the teratogenic effect of elevated Phe levels in poorly controlled PKU during pregnancy is well described,^15^, ^16^, ^25^, ^26^ this effect has rarely been reported when the mother was intellectually and physically normal without any feature that would cause suspicion of PKU as the only feature of classical PKU. Indeed, there are few reported cases that suggest MPKUS might be the first presentation in undiagnosed PKU mothers. Three case reports described three women who were diagnosed with PKU only after a pregnancy with MPKU embryopathy; although one was intellectually delayed, the other two were described as completely normal.^27^, ^28^, ^29^ Additionally, Plana et al, reported 4 women who were unaware that they were affected by PKU but all had mild intellectual defects and two of the mothers had classical PKU phenotypes.^30^

We reported a 32‐year‐old intellectually normal female who was found to have classical PKU in the course of duo exome sequencing performed because of a history of recurrent pregnancy loss and early neonatal deaths with malformations including brain anomalies and congenital heart defect. Despite knowledge that her brother and niece were affected with PKU, she was never suspected of having PKU since she did not express any phenotypic feature, particularly intellectual deficiency. Her son, a six‐year‐old with a developmental delay mainly comprising speech delay, cognitive delay, and behavioral issues, had been investigated with plasma amino acids and exome sequencing which were all unremarkable. It is likely that these features resulted from untreated maternal PKU. Despite, previously reported cases with undiagnosed mothers presented with MPKUS, it is not the typical presentation of PKU. Therefore, the reliance on the phenotypic features of PKU for the suspicion of the disorder in the pregnant woman is not sufficient to always identify MPKUS in time for effective therapy to the mother and avoid teratogenicity.

Children born to mothers with PKU are at increased risk of intellectual disability, microcephaly, congenital heart disease, and intrauterine growth retardation.^14^, ^15^, ^16^ The risk of these abnormalities increases if the Phe concentration exceeds 360 μmol/L and if not optimized early in pregnancy. In addition, spontaneous miscarriage has been reported in up to 30% of pregnancies when maternal Phe exceeds 1000 μmol/L.^14^ Indeed, RZ reported here had two early miscarriages, one of which had normal chromosome analysis (not performed in the first miscarriage). This case emphasizes that undiagnosed maternal phenylketonuria still exists. It is important to be aware of this in normal appearing mothers when an offspring shows features of the MPKUS so as to avoid teratogenicity in future pregnancies. Thus, this report emphasizes expansion of the phenotypic spectrum of classical PKU and the need to evaluate mothers with recurrent pregnancy loss for PKU. As population sequencing becomes more common, the incomplete penetrance and variable expressivity of even well described conditions is increasingly recognized.^31^

## AUTHOR CONTRIBUTIONS

Malak Ali Alghamdi, Anne O'Donnell‐Luria, and Harvey L. Levy conceived and designed the study. Malak Ali Alghamdi and Wajeih Y. AlAali were involved in patient recruitment. Malak Ali Alghamdi, Anne O'Donnell‐Luria, and Harvey L. Levy were involved in manuscript writing and editing. Malak Ali Alghamdi, Naif A. Almontashiri, and Hebatallah H. Ali performed the genetic testing (exome and Sanger sequencing) and the analysis. Malak Ali Alghamdi and Wajeih Y. AlAali performed full clinical characterization of the patient. All authors have read and agreed to the published version of the manuscript.

## CONFLICT OF INTEREST STATEMENT

The authors declare no conflicts of interest.

## ETHICS STATEMENT

Informed consent was obtained from all subjects involved in the study.

## PATIENT CONSENT

Written informed consent to be included in this report was obtained from the participants.

## Data Availability

Data sharing not applicable—no new data generated.
